# Radiographic Results of Single Level Transforaminal Lumbar Interbody Fusion in Degenerative Lumbar Spine Disease: Focusing on Changes of Segmental Lordosis in Fusion Segment

**DOI:** 10.4055/cios.2009.1.4.207

**Published:** 2009-11-25

**Authors:** Sang-Bum Kim, Taek-Soo Jeon, Youn-Moo Heo, Woo-Suk Lee, Jin-Woong Yi, Tae-Kyun Kim, Cheol-Mog Hwang

**Affiliations:** Department of Orthopaedic Surgery, Konyang Universitiy College of Medicine, Daejeon, Korea.; * Department of Radiology, Konyang Universitiy College of Medicine, Daejeon, Korea.

**Keywords:** Lumbar osteoarthritis, Spinal fusion, Transforaminal lumbar interbody fusion, Segmental lordosis

## Abstract

**Background:**

To assess the radiographic results in patients who underwent transforaminal lumbar interbody fusion (TLIF), particularly the changes in segmental lordosis in the fusion segment, whole lumbar lordosis and disc height.

**Methods:**

Twenty six cases of single-level TLIF in degenerative lumbar diseases were analyzed. The changes in segmental lordosis, whole lumbar lordosis, and disc height were evaluated before surgery, after surgery and at the final follow-up.

**Results:**

The segmental lordosis increased significantly after surgery but decreased at the final follow-up. Compared to the preoperative values, the segmental lordosis did not change significantly at the final follow-up. Whole lumbar lordosis at the final follow-up was significantly higher than the preoperative values. The disc height was significantly higher in after surgery than before surgery (*p* = 0.000) and the disc height alter surgery and at the final follow-up was similar.

**Conclusions:**

When performing TLIF, careful surgical techniques and attention are needed to restore and maintain the segmental lordosis at the fusion level.

Transforaminal lumbar interbody fusion (TLIF) is a modified posterior lumbar interbody fusion introduced by Harms and Jeszenszky.1) TLIF was reported to be an effective surgical technique for the treatment of various degenerative lumbar diseases because it allows lateral access to the neural canal. The procedure involves less retraction of the duramater and nerve roots resulting in low complication rates, and produces clinical outcomes and fusion rates similar to those of other techniques.^2^-9) Many studies have focused on maintaining the normal lordotic curve after posterior lumbar interbody fusion combined with instrumentation because the sagittal alignment of a fused segment is associated with both the clinical outcomes and adjacent segment degeneration.^10^-20) Despite the many papers on clinical improvements brought by TLIF, there is a paucity of reports on the radiological changes, particularly the sagittal lordosis after TLIF.21) One study on TLIF performed on patients with isthmic spondylolisthesis described the lordosis of a fused segment after TLIF as being difficult to restore and maintain.21) Therefore, this study examined the sagittal changes, particularly the lordosis of the fused segments as well as the whole lumbar and disc height after TLIF in patients with degenerative lumbar diseases.

## METHODS

**Fig. 1:**
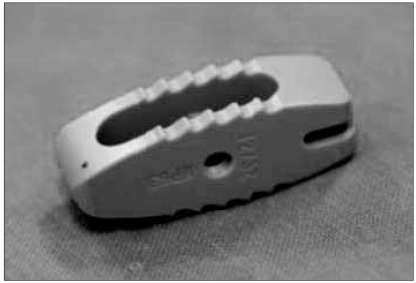
The gross morphology of the cage inserted in the procedure.

**Fig. 2:**
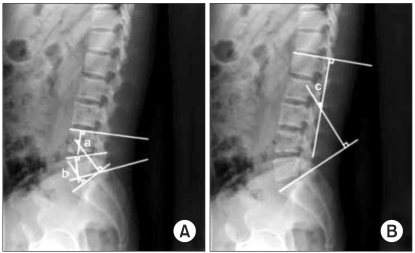
Cobb's angle for segmental lordosis and whole lumbar lordosis. (A) The segmental lordosis (SL) at L4-5 (a) was defined as the angle subtended by the superior endplate line of L4 and the inferior endplate line of L5. The SL at L5-S1 (b) was defined as the angle subtended by the superior endplate line of L5 and superior endplate line of S1. (B) The whole lumbar lordosis (c) was defined as the angle subtended by the superior endplate line of L1 and superior endplate line of S1.

Twenty six patients who had undergone single-level TLIF for degenerative lumbar diseases between February 2005 and February 2007 were reviewed retrospectively. There were 9 males and 17 females with an average age of 55 years (range, 38 to 80 years). The mean follow-up period was 12 months. The preoperative diagnoses were as follows: spondylolisthesis in 12 patients, spinal stenosis in 7, segmental instability in 2, herniation of the intervertebral disc in 3 and a failure of primary surgery in 2 (Table 1). In all cases, biconvex cages with no lordosis (capstone cage®, Medtronics, TN, USA) were used for interbody fusion (Fig. 1) and pedicle screw fixation was performed to create lordosis by compressing the posterior portion. The fused segments were L2-3 in 1, L3-4 in 2, L4-5 in 19 and L5-S1 in 4 patients. All patients were evaluated radiographically before surgery, immediately after surgery, 3 months postoperatively and at the last follow-up visit with particular focus on the fusion success, lordosis of the fused segment and of the whole lumbar and disc height. Interbody fusion was determined to be achieved if a transvertebral osseous bridge had formed anterior and posterior to the cage on the plain radiographs and 3-dimensional CT images, if a radiolucent line between the cage and endplate was not present, if loosening or breakage of pedicle screws did not occur and if there was no motion on the dynamic flexion-extension radiographs. Segmental lordosis (SL) was defined as the angle subtended by the superior endplate line and the inferior endplate line of a segment with an interbody cage. However, the SL at L5-S1 was measured as the angle subtended by the superior endplate line of L5 and the superior endplate line of S1 (Fig. 2A). Whole lumbar lordosis was defined as the Cobb angle formed by the superior endplate line of L1 and superior endplate line of S1 (Fig. 2B). The disc height was determined to be the distance from the midpoint of the anteroposterior diameter of the inferior endplate to the superior endplate. All measurements were performed by a radiologist, who was unaware of the study using picture archiving and communications system (PACS; m-view 5.4; Marotec Medical System, Seoul, Korea). The measurements were performed twice for each parameter with an adequate time interval in order to prevent bias from distorting the results. To minimize the intraobserver error, the average values of the two measurements were used for the evaluation. The average values obtained preoperatively, immediately after surgery, and at the last follow-up were compared and examined for any possible associations between the radiographic parameters. In addition, the relationship between the SL and clinical outcomes was investigated.

The Oswestry Disability Index (ODI) and visual analog scale (VAS) scores of low back pain and radiating pain, which were measured preoperatively, immediately postoperatively and at the last follow-up, were evaluated to assess the clinical outcomes.

Statistical analysis was performed using SPSS ver. 15.0 (SPSS Inc., Chicago, IL, USA). A paired sample t-test with a 99% confidence interval was used to determine the statistical significance of the changes in radiographic values from preoperative, postoperative, to the last follow-up examinations. A partial correlation test was carried out to determine the correlations between the parameters. The association between SL and the clinical symptoms was assessed using a chi-square test and Fisher's exact test.

## RESULTS

Radiographic fusion was achieved in 25 (96.1%) of the 26 patients treated with TLIF. The mean SL preoperatively, immediately after surgery, 3 months postoperatively and at the last follow-up was 14.59 ± 9.81°, 18.21 ± 6.54°, 17.00 ± 7.21° and 16.22 ± 7.39°, respectively. There was a significant difference between the preoperative and immediate postoperative values of SL (*p* = 0.000) but the decreases observed at the last follow-up made the postoperative improvements statistically insignificant (*p* = 0.069). Considering that the differences between the 3 months postoperative and last follow-up periods were statistically significant (*p* = 0.005), the loss of lordosis appeared to have developed after the 3rd postoperative month. The mean whole lumbar lordosis preoperatively, immediately after surgery, 3 months postoperatively and at the last follow-up was 39.10 ± 11.10°, 39.01 ± 8.48°, 42.05 ± 11.77° and 42.55 ± 12.17°, respectively at the last follow-up. The difference between the preoperative and immediate postoperative periods was not significant (*p* = 0.935). On the other hand, remarkable increases in the preoperative values were observed at the 3rd postoperative month (*p* = 0.003) and last follow-up (*p* = 0.000). The mean disc height at the fused segment preoperatively, immediately after surgery, 3 months postoperatively and at the last follow-up was 9.13 ± 2.92 mm, 11.64 ± 1.94 mm, 11.37 ± 1.92 mm and 10.90 ± 2.02 mm, respectively. The increases were significant from before surgery to the last follow-up period (*p* = 0.000). However, the values at the 3rd postoperative month were lower than those obtained immediately after surgery (*p* = 0.01) and slight decreases were also observed at the last follow-up (*p* = 0.002). The mean intraobserver error for the SL, whole lumbar lordosis and disc height was 1.07° ( range, 0 to 3.86°), 2.31° (range, 0.03 to 5.76°) and 0.65 mm (range, 0.03 to 1.85 mm), respectively (Table 2).

**Fig. 3:**
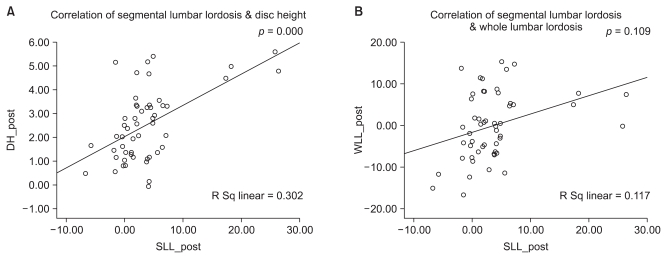
Correlation between both the segmental lordosis and disc height (A) and segmental lordosis and whole lumbar lordosis (B) after surgery. The graph shows a positive correlation between the segmental lordosis and disc height after surgery. However, there was no correlation between the segmental lordosis and whole lumbar lordosis after surgery.

**Fig. 4:**
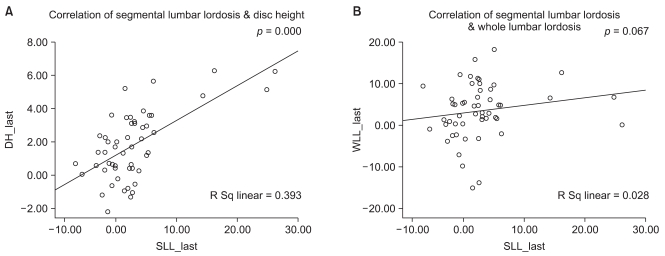
Correlation between both segmental lordosis and disc height (A) and segmental lordosis and whole lumbar lordosis (B) after the final follow-up. The graph shows a positive correlation between the segmental lordosis and disc height after the last follow-up. However, there was no correlation between the segmental lordosis and whole lumbar lordosis after the last follow up.

A change in value is defined as an increase or decrease greater than the mean intraobserver error. With regard to the changes in the SL from before surgery to the postoperative periods in 26 cases, an increase was observed in 16 cases (61.5%), no change was found in 9 cases (34.6%) and a decrease was noted in 1 case (3.8%). A significant loss of SL was observed postoperatively in 12 (46.2%) of the 26 cases and there was no noticeable change in the remaining 14 cases (53.8%). The assessment of the entire lumbar lordosis did not include the immediate postoperative values because the radiographs were not taken with the patient in the standing position. Instead, the values at the 3rd postoperative month were compared with the preoperative ones. Of the 26 cases, an increase was noted in 17 cases (65.4%), no change was observed in 4 cases (15.4%) and a decrease was found in 5 cases (19.2%). Among the 17 cases showing an increase, a loss of lordosis was observed at the last follow-up in 3 cases (17.6%) but the lordosis was maintained or increased until the last follow-up in 18 cases (69.2%) including the remaining 14 cases and other 4 cases showing no changes. A partial correlation test showed that the pre-and postoperative changes in the SL were associated with the disc height (*p* = 0.001) but not with the whole lumbar lordosis (*p* = 0.09) (Fig. 3). The changes in SL between the preoperative and last follow-up periods were also related to the disc height (*p* = 0.000) but not with the whole lumbar lordosis (*p* = 0.067) (Fig. 4).

The association between the SL and clinical symptoms was also examined. In 12 cases showing a loss of SL during the postoperative follow-up, 9 (75%) had remarkable clinical improvement, 1 moderate improvement and 2 poor results. The clinical outcomes appeared better in the 14 cases with no loss of SL observed until the last follow-up: 12 cases (85.7%) exhibited noticeable enhancement, 2 cases showed (14.3%) a moderate outcome. No poor outcomes were observed. However, the association was not statistically significant (*p* = 0.422).

## DISCUSSION

The impact of a lumbar fusion on adjacent segment degeneration has yet to be established but has been the subject of many studies.^10^,^20^,^22^-24) Some authors reported that lumbar fusion resulted in increased mobility and load to the adjacent segments.^10^,^20^,25) Among the various relevant factors, some researchers have described the sagittal alignment of a fused segment as being associated with adjacent segment degeneration.^20^,^23^-25) According to the cadaveric study by Umehara et al.,20) loading of the posterior column of the adjacent segments increased with decreasing lordosis of a fused segment. Oda et al.25) reported that kyphotic fusion might lead to degenerative changes in the adjacent facet joints based on their animal model study. In a cadaveric study on the association between the lordosis of a fused segment and the adjacent segment motion, Akamaru et al.10) reported that hypolordotic alignment of the fused segments caused the greatest amount of flexion-extension motion at the superior adjacent segment, and suggested that this was related to the degeneration of the adjacent segments, emphasizing the importance of maintaining lordosis in lumbar interbody fusion of L4-L5. Against this background, many posterior lumbar interbody fusion techniques using a variety of cages have been introduced to minimize the adjacent segment degeneration by restoring the normal sagittal alignment.^11^-19)

TLIF, which was reported to have better surgical outcomes and fewer complications than posterior lumbar interbody fusion, has recently become the preferred procedure and has become one of the minimally invasive fusion techniques with the invention of percutaneous pedicle screw fixation. The two procedures were also compared in a previous study. Patients with spondylolisthesis at one-level were divided into two groups according to the surgical technique: bilateral posterior lumbar interbody fusion and unilateral TLIF. Satisfactory clinical outcomes and radiographic fusion rates were obtained in both groups with no remarkable differences. TLIF had fewer complications and less operative time that with the other technique, even though no statistical significance was found.26) Many studies have reported the efficacy of TLIF in terms of clinical outcomes,^2^-^9^,26) but radiographic changes in the disc height and sagittal lordosis have rarely been included in these studies. Kwon et al.21) measured the slip angle at the fused segment on the sagittal plane in patients with isthmic spondylolisthesis who underwent TLIF and reported that the surgical procedure resulted in an increase in disc height and the restoration of anterolisthesis, but the slip angle was not altered and no kyphotic deformity developed in many cases. According to them, the factors associated with a kyphotic deformity at the fused segment were as follows: distraction required after pedicle screw insertion can result in a mild kyphotic deformity; and relatively posterior location of the cage is unavoidable with TLIF, which can make it hard to achieve a normal lordosis of the fused segment even under compression. They suggested that a restoration of normal lordosis could be obtained by inserting a small cage as anteriorly as possible and applying compression. In another study, the authors performed TLIFs using 8° wedge cages to restore the normal lordosis.13) However, their procedure was quite different from normal traditional TLIF because two cages were inserted anteriorly via the bilateral approach. Therefore, it should be considered to be posterior lumbar interbody fusion. Moreover, the use of 8° wedge cages cannot be regarded as a breakthrough because it is not a rarity with the posterior approach.

With our procedure, SL, which increased significantly after surgery, decreased gradually during the follow-up to the point of insignificance at the last examination. This gradual loss of lordosis was attributed to three causes. The first is cage subsidence, which can occur when the integrity of the subchondral bone is not preserved during the process of removing the cartilaginous endplate. Reamers are commonly used for distraction of the intervertebral disc space and the removal of the cartilaginous endplate. Unnecessary bone loss is unavoidable considering the concave curvature of the cartilaginous endplate and the square shaped reamers. this is especially so in patients with preoperative symptoms, such as kyphotic deformity and instability resulting from anterior destruction of the vertebral bodies. In addition, cage subsidence appears to be more likely because most patients also present with osteoporosis. Second, the procedures carried out during TLIF may have been responsible. Compared to the posterior approach, which allows parallel insertion of two cages, unilateral TLIFs, in which the cages are placed diagonally, lack anterior support leading to a loss of lordosis. Third, the shape of the cages may have been associated. The cages used in this study were convex in the middle and had no lordosis. Accordingly, the anterior height of the cages was less than the 4° or 8° wedge-shaped cages, which made it difficult to recreate or maintain the lordosis of a fused segment and prevent cage subsidence.

Based on the results of the partial correlation test, a loss of SL and decrease in disc height resulting from cage subsidence appeared to be involved. Meanwhile, no significant changes were noted in the whole lumbar lordosis from the preoperative to immediately postoperative periods. This was attributed to the patient's position during plain radiography, supine not the standing position. However, since the whole lumbar lordosis improved 3 months after surgery and at the last follow-up, it is believed that it was not affected by the loss of SL and adjacent segment hypermobility occurred.

Although no correlation was established between the loss of SL and short-term clinical results in this study, the loss of lordosis of a fused segment or kyphotic deformity may be associated with adjacent segment degeneration in the long-term. Therefore, it is believed that care and attention should be given to introduce lordosis in the normal range as much as possible. For this purpose, it is recommend that a wedge-shaped cage with a large lordotic angle be used in TLIF, and the cage be inserted as anterior as possible after densely impacting the local bone anterior to the cage to restore lordosis. In addition, in order to minimize the subchondral bone loss in the process of cartilaginous endplate removal, the patient's interbody disc shape should be considered when choosing or devising the shape of a reamer.

This study had 2 limitations. First, it was difficult to expect high reliability on the correlation test results involving the clinical symptoms because this study was a retrospective one with only a small number of patients. Second, the 12 months follow-up period was too short to examine the hypermobility and degeneration of the adjacent segments. Therefore, more studies will be needed to address these limitations.

TLIF is a procedure commonly used to treat degenerative lumbar disorders but requires careful consideration of the surgical techniques and equipment because normal lordosis of a fused segment is not difficult to maintain. Studies examining sagittal lordosis of a fused segment and adjacent segment degeneration after a long-term period are currently underway.

## Figures and Tables

**Table 1:**
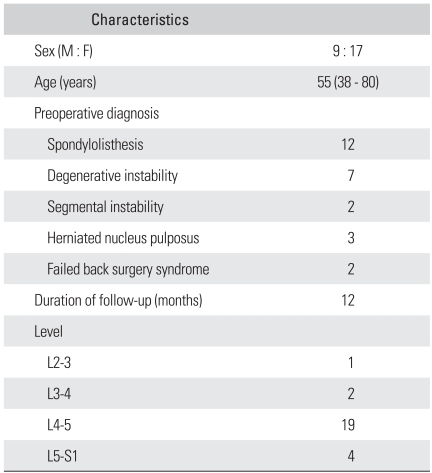
Patients' Dermographics (N = 26)

**Table 2:**
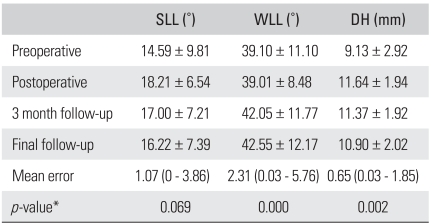
Changes in the Radiographic Parameters Affecting the Sagittal Balance (Mean ± SD)

SLL: Segmental lumbar lordosis, WLL: Whole lumbar lordosis, DH: Disc height. ^*^The mean values for each parameter obtained before surgery and at the last follow-up were compared using a paired sample t-test.
